# Effect of *Saccharomyces uvarum* on lipid oxidation and carbonyl compounds in silver carp mince during cold storage

**DOI:** 10.1002/fsn3.1101

**Published:** 2019-06-25

**Authors:** Lina Xu, Yu Luo, Xiangjin Fu, Feijun Luo, Youzhi Xu, Shuguo Sun

**Affiliations:** ^1^ College of Food Science and Engineering Central South University of Forestry and Technology Changsha China

**Keywords:** carbonyl compounds, docosahexaenoic acid (DHA), eicosapentaenoic acid (EPA), flavor, lipid oxidation, *Saccharomyces uvarum*, silver carp

## Abstract

Fish lipid is highly susceptible to oxidation, resulting in accumulation of toxic substances reactive carbonyl compounds (RCCs), the reduction of nutritional value, and the production of odorous substances. In this study, the effect of yeast (*Saccharomyces uvarum*) on RCCs, fat acid composition, volatiles, and sensory traits in silver carp mince stored at 4°C was evaluated. Yeast eliminated malondialdehyde, 4‐hydroxyl‐2‐hexenal, and 4‐hydroxyl‐2‐nonenal by about 80%, 68%, and 60%, which increased by about 170%, 340%, and 300% in the control, respectively. Yeast helped retain about 80% of the polyunsaturated fatty acids eicosapentaenoic acid (EPA) and docosahexaenoic acid (DHA), respectively; only about 53% and 46% of EPA and DHA, respectively, were maintained in the control. Yeast removed off‐odors hexanal, nonanal, and decenal, resulting in enhanced sensory traits. These findings were economically important for improving the quality of fish products. It might present an approach to improve the flavor of fish products.

## 1 INTRODUCTION

Silver carp (*Hypophthalmichthys molitrix*) has worldwide importance because of its huge annual output and high nutritional value (rich in protein and polyunsaturated fatty acids, PUFAs) (Li, Sinclair, & Li, 2011). In addition, carps found in fresh water in the United States, such as the Mississippi River, have recently drawn increase attention for their potential as a protein resource for the growing global population (Taskaya, Chen, & Jaczynski, 2010).

However, silver carp is highly susceptible to lipid oxidation because of its relatively high polyunsaturated fatty acids (PUFA) content (Fu et al., 2012; Fu, Lin, Xu, & Wang, 2015). Lipid oxidation decreases the nutritional value of lipids and subsequently leads to the development of fishy off‐odor and rancid taste (Farvin, Grejsen, & Jacobsen, 2012), also resulting in the accumulation of toxic reactive carbonyl compounds (RCCs) (Sakai, Matsushita, Sugamoto, Matsushita, & Sugamoto, 1997). Fishy off‐odor and rancid taste are due to secondary lipid oxidation products, which mostly include carbonyl compounds, including hexanal, nonanal, and decanal (Fu, Xu, & Wang, 2009). These volatiles have considerably low aroma threshold and have been served as indicators of lipid rancidity in food (Lehto, Laakso, & Lehtinen, 2003). RCCs, such as malondialdehyde (MDA), 4‐hydroxyl‐2‐hexenal (HHE), and 4‐ hydroxyl‐2‐nonenal (HNE), are readily diffused into the cellular media where they may exert toxicological effects by reacting with critical bio‐molecules in vivo (Lynch & Faustman, 2000). RCCs are absorbed in the intestines. They not only directly damage the intestine but enter the blood circulation as well, harming the liver, kidney, lung, and other organs (Steppeler, Haugen, RΦdbotten, Haugen, & RΦdbotten, R., 2016).

RCCs were detected in cold stored fish, pork, and beef (Sakai et al., 1997). The RCCs’ content in these products increased significantly during cold storage. HHE’s content in fresh carp muscle varied from 1128.4 μg/kg to 4873.2 μg/kg (Sakai et al., 1997). HHE’s content in yellow herring fillets frozen at −20°C increased from 374.48 ± 238.08 μg/kg to 7541.68 ± 544.36 μg/kg in 28 weeks. Fresh saury contained 1698.8 ± 793.6 μg/kg of HHE, which increased to about 6,200 μg/kg during frozen storage at −20°C for 3 months and increased to 12,000 μg/kg during storage for 12 months (Tanaka et al., 2013). The threshold of toxicological concern (TTC) of HHE and HNE in fish meat is about 1800 μg/kg (for an adult weight of 60 kg and average fish meat intake of 50 g/d) (Papastergiadis et al., 2014); thus, the RCCs’ content in a significant portion of fish products is considerably higher than TTC, and their risks of food safety need to be considered.

Numerous technological approaches have been adopted to reduce lipid oxidation in fish products (Song, Liu, Shen, Liu, Shen, You, & Luo, 2011). The use of synthetic or natural antioxidants is one of the most applied employed measures (Shi, Cui, Yin, Cui, Yin, Luo, & Zhou, 2014). However, owing to growing consumer concerns over the possible carcinogenic effects of chemical preservatives, interest in the development of bio‐preservation techniques has increased, including the use of natural or controlled microflora to extend the shelf life and improve the quality of foods (Devlieghere, Vermeiren, & Debevere, 2004). *Lactobacillus* and yeast are the most attractive microflora.

In several reports, *Lactobacillus* and yeast are characterized by their outstanding antioxidant activity (Chen et al., 2010; Kakuta et al., 1999; Vieira, Melo, & Ferreira, 2017). Baka found that *L. sakei* 4,413 could effectively inhibit lipid oxidation in sausages and reduce the thiobarbituric acid reactive substances (TBARS) by about 1 mg/kg relative to that of the blank control (Baka, Papavergou, Pragalaki, Bloukas, & Kotzekidou, 2011). Zeng, Xia, Jiang, Xia, Jiang, and Yang (2013) indicated that *Lactobacillus plantarum* 120, *Pediococcus pentosaceus* 220, and *Saccharomyces cerevisiae* 22 could effectively inhibit lipid oxidation during fermentation of “Suan Yu,” a traditional fermented fish product characterized by a sour taste. The yeast strain of *Kluyveromyces marxianus KU140723‐02, S. cerevisiae ATCC6037*, and *Kluyveromyces lactis ATCC34440* screened from kefir exhibited high antioxidant activity (Cho et al., 2018). Moreover, the Chinese traditional fish product “Zao‐yu” fermented with yeast (*S. cerevisiae*) had a floral and fruity aroma. Its fishy and rancid off‐odor were inhibited or removed. As previously mentioned, the off‐odor of silver carp mince was mainly caused by aldehydes, and yeast typically contains high‐activity aldehyde dehydrogenase/reductase, which transforms aldehydes into corresponding acids/alcohol with a lower odor activity (Datta, Annapore, & Timson, 2017; Wang, Xiao, et al., 2017). Another advantage of yeast strains over *Lactobacillus* is that yeast is easier to culture.

We compared several strains, including *Lactobacillus delbrueckii* subsp. bulgaricus, *Streptococcus thermophilus*, *S. cerevisiae,* and *S. uvarum*, about their activity of aldehyde dehydrogenase; the results showed that the *S. uvarum* had the highest activity (data not shown). Thus, we assumed that yeast *S. uvarum* could be a good bio‐preservative for reducing lipid oxidation, removing RCCs, and inhibiting the production of off‐odor in silver carp mince during storage. So the present study evaluates the effect of *S. uvarum* on preventing lipid in silver carp mince during cold storage from oxidation. The MDA, HHE, HNE, and fatty acid components were assayed. Gas chromatography–mass spectrometry (GC–MS) was carried out to detect volatile compounds. Sensory evaluation was further conducted, in order to help develop a new bio‐preservative for fish mice.

## 2 MATERIALS AND METHODS

### 2.1 Preparation of silver carp mince

Live silver carp (average weight of about 2 kg/fish) was purchased from a local fishery market. The fishes were killed by knocking them on top of the head using a wooden club, and then, the fish was gutted, headed, skinned, and washed (Qin et al., 2016). Mince samples were collected manually and stored at 4°C immediately.

### 2.2 Preparation of yeast cell

Yeast (*S. uvarum*) was purchased from Angel yeast Ltd. (Hubei, China). Yeast was cultured in medium (0.1% [w/v] yeast extract, 0.2% [w/v] peptone, 5% [w/v] glucose) at 28°C. After 48 hr of growth, the cells were harvested by centrifugation for 10 min at 5,000 × *g* and 4°C (4K15, Sigma).

### 2.3 Effect of yeast on lipid oxidation in silver carp mince

Wet yeast cell was carefully and evenly mixed with the fish mince. About 10^7^ cfu/g mince of wet yeast cell was added. The yeast‐treated sample (YTM) and the blank control samples (BCM) were packed in polyvinyl chloride bags and then stored in refrigerators at 4°C. Samples were taken randomly for analysis at selected time intervals (0, 24, 48, and 72 hr).

### 2.4 Determination of MDA

The MDA was assessed in accordance with the literature (Steppeler et al., 2016), using thiobarbituric acid as chromogenic agent, quantified the chromophore at 532 nm. Standard curve was established using 1,1,3,3‐tetraethoxypropane (Sigma).

### 2.5 Determination of HHE and HNE

HHE and HNE were measured in accordance with the literature (Steppeler et al., 2016; Surh & Kwon, 2005) and little modification. 18 ml of 0.1% ascorbic acid solution (dissolved in water) was added to the sample, homogenized at 11,000 rpm for 2 min, and centrifuged (10,000 × *g* for 10 min at 4°C), and the supernatant was collected. The extraction was repeated analogously, combined the supernatant. Then, the dichloromethane (10 ml) was used to extract for three times, and the organic phase was collected after centrifuged at 10,000 *g* for 2 min at 4°C, then filtered (Whatman, 595 1/2), and dried at 30°C with nitrogen. 200 μl of BSTFA (N,O‐bis(trimethylsilyl) trifluoroacetamide) (Sigma) was added and kept at room temperature (25°C) for 3 hr to derivatize (Steppeler et al., 2016).

After derivatization, 1 μl of solution was analyzed with a gas chromatography on a capillary column (SH‐Rtx‐5 SIL MS, 30 m × 0.25 mm × 0.25 μm, SHIMADZU) with a MS detector (GCMS‐QP2010 Ultra, SHIMADZU). The parameters were set as follows: splitless mode, injector temperature 250°C, carrier gas (Helium) constant flow of 1.0 ml/min, transfer line temperature 280°C, ion source 230°C, EI 70 eV, and oven program: 100°C held for 4 min, increased at 15°C/min to 300°C, and then kept at 300°C for 3 min. Fragment ions with m/z of 157, 186 and m/z of 157, 199 were monitored for HHE and HNE in selective ion monitoring (SIM), respectively (Surh & Kwon, 2005). Used external standard methods to quantify with the same derivatization procedure. The standard HHE and HNE was purchased from Cayman Chemical Co.

### 2.6 Analysis of fat acid composition

About 5 g of each sample was homogenized for lipid extraction, using chloroform–methanol (2:1, v/v) (Fu et al., 2015). The extracted oil was analyzed for methyl esters of fatty acids by gas chromatography (GCMS‐QP2010 Ultra, SHIMADZU) on a capillary column (SH‐Rtx‐5 SIL MS, 30 m × 0.25 mm × 0.25 μm, SHIMADZU) (Fu et al., 2015). The fat acids were quantified using external standard methods (the standards were purchased from Sigma).

### 2.7 Solid‐phase microextraction (SPME) GC–MS for volatiles analysis

One gram mince was put into a 15 ml sample bottle (Supelco), then added 2 ml distilled water and 0.5 g NaCl, followed by SPME immediately. The SPME fiber used was divinylbenzene/Carboxen/poly(dimethyl‐siloxane) (DVB/Carboxen/PDMS) (50/30 μm, 2 cm) (Supelco), exposure time was 60 min, and temperature was 25°C (Fu et al., 2009).

GC–MS was conducted in a gas chromatography system equipped with a SH‐Rtx‐5 SIL MS capillary column and an electron ionization ion source operated at 70 eV. Temperature programming was applied from 35°C to 220°C at a rate of 10°C/min. The volatile compounds (hexanal, octanal, nonanal, decenal, 1‐hexanol, 1‐octen‐3‐ol, and 2‐ethyl‐1‐hexanol) were quantified using external standard methods (the standards were purchased from Sigma). The results are the means of three measurements (standard deviation <8%).

### 2.8 Sensory evaluation

The mince samples were stored in 100 ml brown bottles with caps for sensory evaluation. Ten trained panelists sniffed the samples, using a scale of 0–10 (with 10 being the strongest) to evaluate the odors (Richards & Hultin, 2000). The average was defined as the final score. In training sessions, assessors smelled the mince, discussing among themselves the most appropriate descriptions and agreeing on the meaning of their elected descriptors (Table 1).

**Table 1:** Descriptive odor traits used in sensory evaluation and olfactometry

Grassy	Associated with fresh grass
Oxidized oil	Associated with rancid pork fat
Fishy	Associated with chopped silver carp mince stand at 25°C for 0.5 hr
Sweet–fruity	Associated with fresh fruit
Mushroom‐like	Associated with fresh mushroom

### 2.9 Statistical analysis

The data represent the mean and standard deviation from three independent experiments. The statistical significance of data was determined using ANOVA in SAS ver. 6.0 (SAS Institute Inc.).

## 3 RESULTS

### 3.1 Effect of yeast on MDA, HHE, and HNE contents in silver carp mince

**Figure 1:**
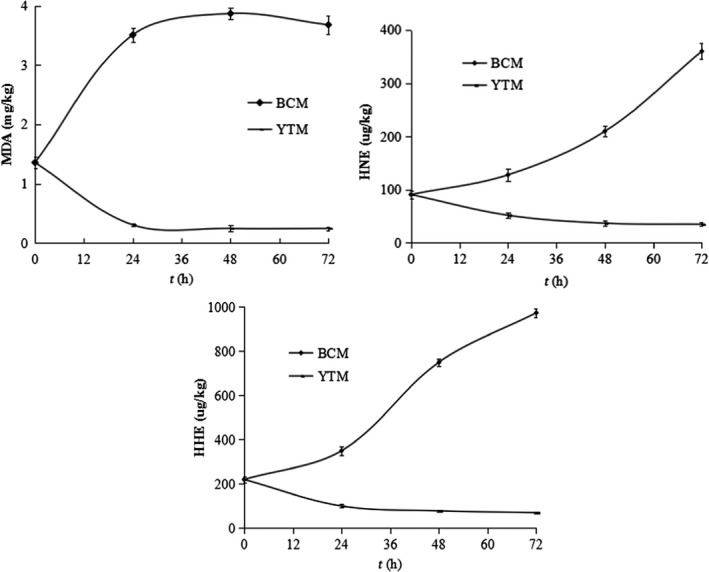
Effect of yeast on MDA, HHE, and HNE contents in silver carp mince during cold storage (*n* = 3). BCM and YTM represent blank control mince and yeast‐treated mince, respectively

The MDA is believed to be characteristic decomposition product of unsaturated fatty acid oxidation. During cold storage of the mince, the MDA content of the control (BCM) increased rapidly in 24 hr from 1.36 ± 0.10 mg/kg to 3.51 ± 0.12 mg/kg, indicating the occurrence of serious lipid oxidation (Figure 1). However, the MDA content of the yeast‐treated mince (YTM) was significantly lower than that of BCM (*p* < 0.05). Notably, yeast markedly decreased the MDA content in the first 24 hr from 1.36 ± 0.10 mg/kg to 0.31 ± 0.02 mg/kg and then almost remained constant at this low level (Figure 1). The HHE and HNE contents in BCM increased rapidly, from 220.3 ± 13.5 μg/kg and 91.2 ± 7.1 μg/kg to 974.5 ± 19.8 μg/kg and 360.4 ± 15.2 μg/kg, respectively. The HHE and HNE contents in YTM decreased throughout the period of storage and were significantly lower than those of the control (*p* < 0.05). In other words, the yeast eliminated MDA, HHE, and HNE by about 80%, 68%, and 60%, which increased by about 170%, 340%, and 300% in the control, respectively.

### 3.2 Effect of yeast on the fatty acid composition of silver carp mince

Fresh silver carp mince contains 60.21 g/100 g unsaturated fatty acids. EPA (20:5) and DHA (22:6), comprising about 9.20 g/100 g fat, which is almost equal to that of sea water fish, indicate the high nutritional value of silver carp. Unsaturated fatty acid in silver carp mince, particularly PUFA, significantly decreased during cold storage (Table 2). The palmitoleic acid, oleic acid, linoleic acid, linolenic acid, arachidonic acid, EPA, and DHA in the control decreased by 36.01%, 34.85%, 34.76%, 37.84%, 48.25%, 46.35%, and 53.26%, respectively, during cold storage for 3 day. Yeast effectively helped retain these FAs. Palmitoleic acid, oleic acid, linoleic acid, linolenic acid, arachidonic acid, EPA, and DHA decreased by only 14.90%, 13.73%, 13.57%, 24.64%, 24.53%, 21.42%, and 22.99%, respectively (*p* < 0.05). In summary, after 3 day of cold storage, only about 50% of EPA and DHA in silver carp mince remained, whereas with yeast treatment, about 80% remained. These results were similar to Wang, Xiao, et al. (2017)**)** report; they found that hairtail fish fermented with *L. plantarum* and *P. pentosaceus* contained 23% more PUFA and 25% more MU

**Table 2:** Effect of yeast on the fatty acid composition (g/100 g fat) of silver carp mince during cold storage

	Fresh mince	BCM‐1	BCM‐2	BCM‐3	YTM‐1	YTM‐2	YTM‐3
Palmitoleic acid	8.72 ± 0.18^ab^	7.90 ± 0.15^c^	6.35 ± 0.35^e^	5.58 ± 0.10^f^	9.33 ± 0.32^a^	8.76 ± 0.25^b^	7.42 ± 0.26^d^
Oleic acid	24.25 ± 0.92^a^	20.23 ± 0.80^bc^	18.87 ± 0.80^c^	15.80 ± 0.95^d^	23.31 ± 0.78^a^	21.19 ± 0.98^b^	20.92 ± 0.75^b^
Linoleic acid	4.20 ± 0.13^b^	4.10 ± 0.11^b^	3.29 ± 0.08^d^	2.74 ± 0.13^e^	4.56 ± 0.10^a^	4.09 ± 0.10^b^	3.63 ± 0.09^c^
Linolenic acid	9.17 ± 0.17^a^	7.98 ± 0.10^c^	6.43 ± 0.12^f^	5.70 ± 0.22^g^	8.86 ± 0.10^b^	7.49 ± 0.12^d^	6.91 ± 0.15^e^
Arachidonic acid	3.71 ± 0.10^a^	3.10 ± 0.08^d^	2.86 ± 0.10^c^	1.92 ± 0.03^e^	3.43 ± 0.20^b^	3.02 ± 0.08^cd^	2.80 ± 0.10^c^
EPA	6.58 ± 0.14^a^	5.64 ± 0.08^c^	4.98 ± 0.09^d^	3.53 ± 0.06^e^	6.14 ± 0.07^b^	5.74 ± 0.15^c^	5.17 ± 0.08^d^
DHA	2.61 ± 0.06^a^	2.12 ± 0.05^c^	1.76 ± 0.05^d^	1.22 ± 0.06^e^	2.56 ± 0.08^a^	2.41 ± 0.05^b^	2.01 ± 0.06^c^

Note

BCM‐1, BCM‐2, and BCM‐3, representing blank control mince stored at 4°C for 1, 2, and 3 days, respectively. YTM‐1, YTM‐2, and YTM‐3, representing yeast‐treated mince stored at 4°C for 1, 2, and 3 days, respectively. Means in each row having different superscript letters are significantly different (*p* < 0.05) (*n* = 3).

FA than that of the control (*p* < 0.05), mostly resulting from the capability of *Lactobacillus* to inhibit the oxidation of PUFA and the growth of *Pseudomonas* with high lipoxygenase activity.

### 3.3 Effect of yeast on volatile compounds in silver carp mince

The contents of seven key volatile compounds, namely hexanal, octanal, nonanal, decenal, 1‐hexanol, 1‐octen‐3‐ol, and 2‐ethyl‐1‐hexanol (Table 3), were determined to evaluate the effects of yeast treatment. Octanal, hexanal, nonanal, decenal, 1‐hexanol, 1‐octen‐3‐ol, and 2‐ethyl‐1‐hexanol were identified as the characteristic volatile compounds of silver carp (Zhou, Chong, Ding, Chong, Ding, Gu, & Liu, 2016). Their odors were described as grassy, fishy and grassy, green and fatty, oxidized oil and fishy, grassy, mushroom‐like, and sweet–fruity, respectively (Fu et al., 2009). In addition, hexanal was used as an oxidation marker of n‐6 FA, correlated with rancid odor and off‐odor (Albertos, Gringer, Rico, Gringer, Rico, & Baron, 2016). 2‐Ethyl‐1‐hexanol was determined as a characteristic volatile product of yeast (Geng, Xia, Zu, Xia, & Zu, 2014).

**Table 3:** Effect of yeast on the content of volatile compounds (μg/kg) in silver carp mince during cold storage

	Fresh mince	BCM‐1	BCM‐2	BCM‐3	YTM‐1	YTM‐2	YTM‐3	Threshold^a^
1‐Hexanol	18.5 ± 1.4^e^	75.2 ± 5.3^d^	375.4 ± 13.8^b^	521.9 ± 60.7^a^	40.2 ± 5.4^de^	90.6 ± 7.3^d^	186.8 ± 6.9^c^	9
1‐Octen−3‐ol	4.8 ± 0.4^d^	9.1 ± 0.6^c^	12.5 ± 0.9^b^	21.6 ± 1.0^a^	5.2 ± 0.6^d^	6.1 ± 0.5^d^	9.0 ± 0.5^c^	1
2‐Ethyl−1‐hexanol	99.3 ± 8.1^f^	140.5 ± 10.4^f^	199.2 ± 11.6^e^	317.4 ± 12.2^c^	262.8 ± 8.5^d^	925.6 ± 38.0^b^	1566.9 ± 60.3^a^	300
Hexanal	14.5 ± 0.9^d^	28.6 ± 1.2^c^	70.9 ± 3.2^b^	106.8 ± 5.1^a^	2.5 ± 0.3^e^	1.5 ± 0.4^e^	nd.	5
Octanal	2.4 ± 0.5^d^	4.1 ± 0.6^c^	5.6 ± 0.4^b^	18.3 ± 0.9^a^	3.3 ± 0.5^cd^	4.2 ± 0.5^c^	4.5 ± 0.6^c^	3
Nonanal	2.2 ± 0.3^c^	2.9 ± 0.4^b^	3.4 ± 0.5^b^	4.1 ± 0.4^a^	9.0 ± 0.3^d^	nd.	nd.	1
Decanal	0.5 ± 0.2^c^	2.2 ± 0.3^b^	2.5 ± 0.5^b^	4.7 ± 0.2^a^	0.6 ± 0.3^c^	0.4 ± 0.2^c^	nd.	0.1

Note

BCM‐1, BCM‐2, and BCM‐3, representing blank control mince stored at 4°C for 1, 2, and 3 days, respectively. YTM‐1, YTM‐2, and YTM‐3, representing yeast‐treated mince stored at 4°C for 1, 2, and 3 days, respectively. Means in each row having different superscript letters are significantly different (*p* < 0.05) (*n* = 3).

a Odor thresholds were mainly obtained from the literature and an online database: Zhu, Wang, Xiao, Wang, Xiao, & Niu, 2018; http://www.odour.org.uk/odour/index.html.

Comparison between the control and the samples treated with yeast revealed large differences in the intensities of all compounds. The intensities of all off‐odor aldehydes were reduced by yeast except for octanal. The content of hexanal, nonenal, and decanal was below the minimum limit of detection in YTM after cold storage for 3 day; meanwhile, their contents in BCM increased by about 6.3, 0.8, and 2.1 times, respectively. Its fishy off‐odor presents a disadvantage for silver carp and thus should be removed by yeast. Wang also reported that fermentation with *L. plantarum* and *P. pentosaceus* significantly decreased the hexanal content of dry‐cured hairtail (*p* < 0.05) (Wang, Xiao, et al., 2017). The octanal contents in BCM and YTM were increased by about 6.5 and 0.8 times, respectively. The 1‐hexanol in BCM and YTM was increased by about 28 and 9 times, respectively. The 1‐octen‐3‐ol contents in BCM and YTM were increased by about 4.7 and 1.4 times, respectively. The 2‐ethyl‐1‐hexanol contents in BCM and YTM were increased about 2.2 and 14.8 times, respectively.

### 3.4 Effect of yeast on the sensory traits of silver carp mince

**Figure 2:**
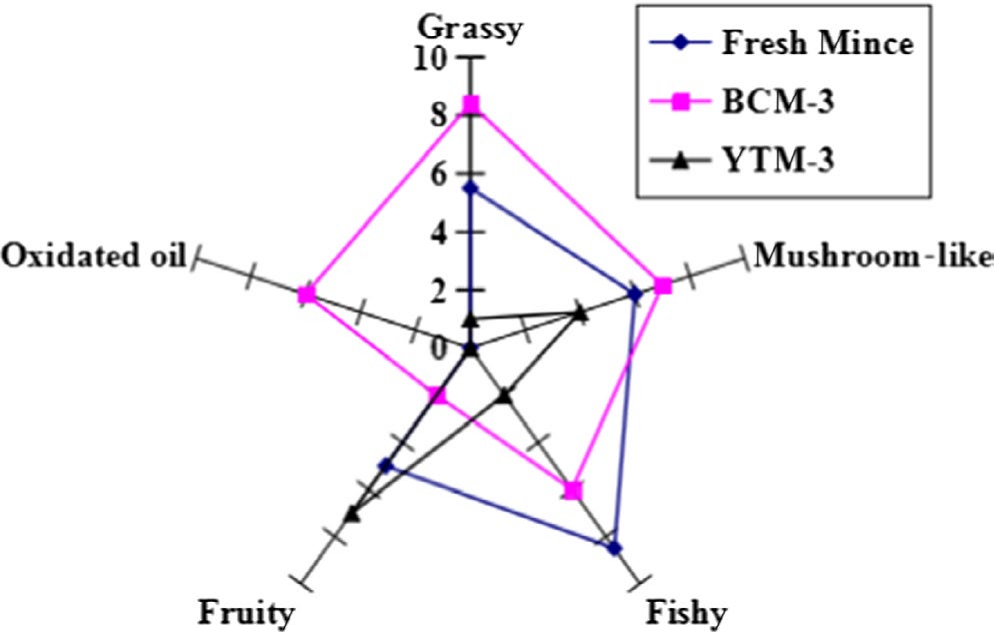
Effect of yeast on the sensory traits of silver carp mince. BCM and YTM represent blank control mince and yeast‐treated mince, respectively

The sensory traits of fresh silver carp mince were identified as mild–delicate grassy, mushroom‐like, and sweet–fruity (Figure 2), which is consistent with the results obtained by Fu et al. (2015) and Zhou et al. (2016). The control mince (BCM) in cold storage for 3 days was observed as oxidized oil and fishy in odor, whereas the yeast‐treated mince (YTM) was noted as slightly sweet–fruity.

During cold storage, the strength of grassy, fishy, oxidized oil‐like, and mushroom‐like odors of the control mince increased, whereas the sweet–fruity flavor decreased. With yeast treatment, the strength of grassy, fishy, mushroom‐like, and oxidized oil odors was decreased, while the sweet–fruity aroma was increased. Combined with the results of volatile compounds (Table 3), changes in the strength of grassy, fishy, and oxidized oil odors were related to the change in the content of hexanal, nonanal, and decenal. The enhanced sweet–fruity aroma in YTM might be attributed to the accumulation of 2‐ethyl‐1‐hexanol. The lower score of YTM with respect to rancid odors (grassy, fishy, and oxidized oil‐like) coincides with the lower levels of lipid oxidation‐derived components. Zhou et al. (2016) found washing removed off‐odor compounds in silver carp surimi effectively, resulting in improved flavor. The present results show that yeast could not only reduce the off‐odor volatile but increase the content of aroma substances as well.

## 4 DISCUSSION

Several studies evaluated the inhibitory effect of antioxidants on the accumulation of RCCs. For instance, 0.6% VE could significantly inhibit increases in MDA and HHE contents in frozen saury (Tanaka et al., 2013). Another report found that for ice‐stored yellow herring, cherry vinegar inhibited the accumulation of MDA but promoted the accumulation of HHE (1% cherry vinegar was added; stored on ice for 7 day; HHE increased from 2.108 ± 0.744 μg/kg to 8456.8 ± 747.72 μg/kg; the blank control was 204.6 ± 23.56 μg/kg; MDA only increased from 37.44 ± 3.60 μg/kg to 93.60 ± 8.64 μg/kg; and MDA in blank control was 172.08 ± 7.92 μg/kg) (Munasinghe et al., 2003). Yao et al. (2014) found that the water‐soluble material of yeast could effectively decrease the MDA content in the intestinal mucosal cell culture (grass carp, *Ctenopharyngodon idella*), thereby protecting the cells from damage caused by MDA. However, as far as we know, no report has been conducted on the use of yeast cell to inhibit or remove MDA, HHE, and HNE in fish mince.

Yeast exhibited good antioxidant activity. However, the difference in the capability of yeast to decrease MDA, HHE, HNE, hexanal, nonanal, and decanal with a simple inhibitor (Figure 1 and Table 3) suggested yeast strongly exhibited the capacity to react and/or convert carbonyl compounds to other compounds. Thus, an enzyme mechanism was assumed. Carbonyl compounds transforming enzymes include alcohol dehydrogenase (ADH), aldehyde dehydrogenase (ALDH), aldehyde oxidase (AOX), and aldehyde deformylating oxygenase (ADO). ADH catalyzes the reduction of aldehydes; ALDH catalyzes the irreversible oxidation of aldehydes into their corresponding carboxylic acids; and AOX and ADO convert aldehydes into corresponding acids and alkanes, respectively (Foo, Susanto, Keasling, Susanto, & Keasling, 2017). ADH and ALDH have been purified from yeast (Datta et al., 2017; Wang, Wu, & Li, 2017). Yeast can efficiently perform chemo‐, regio‐ and stereo‐selective bio‐transformation of alcohols and aldehydes, affording the corresponding carboxylic acids (Svitel & Sturdik, 1995). Yeast involved in the removal of a wide range of undesirable wort carbonyl compounds during fermentation (Iersel, Brouwer‐Post, Rombouts, Brouwer–Post, Rombouts, & Abee, 2000). “Green note” aldehydes were successfully reduced into their corresponding alcohols by commercial yeast ADH and yeast cells (Fauconnier et al., 1999). Furthermore, Lehto et al. (2003) reported that the ALDH‐type activity present in oat efficiently decreases hexanal content and prevents the accumulation of short chain aldehydes that cause off‐odors. ALDH purified from bovine liver mitochondria was used to remove the “green” odor of soybean products (Sawada, Hara, Nakayama, Hara, & Nakayama, 1982). As far as we know, no studies on the enzymatic conversion of carbonyl compounds in muscle foods have been reported.

## 5 CONCLUSIONS

Lipid oxidation in silver carp mince during cold storage was effectively inhibited by yeast (*S. uvarum*). Yeast (*S. uvarum*) decreased MDA, HHE, and HNE and helped retain PUFA. It also removed most of the off‐odor compounds. The *S. uvarum* might be a good bio‐preserver for fish mince. These findings were significant because of the economic value of improving the quality of fish products.

## CONFLICT OF INTEREST

The authors declare that they do not have any conflict of interest.

## ETHICAL APPROVAL

This study does not involve any human or animal testing.

## References

[fsn31101-bib-0001] Albertos, I. , Gringer, N. , Rico, D. , & Baron, C. P. (2016). Salted herring brine as a coating or additive for herring (*Clupea harengus*) products ‐A source of natural antioxidants? Innovative Food Science and Emerging Technologies, 37, 286–292. 10.1016/j.ifset.2016.09.008

[fsn31101-bib-0002] Baka, A. M. , Papavergou, E. J. , Pragalaki, T. , Bloukas, J. G. , & Kotzekidou, P. (2011). Effect of selected autochthonous starter cultures on processing and quality characteristics of Greek fermented sausages. LWT‐ Food Science and Technology, 44(1), 54–61. 10.1016/j.lwt.2010.05.019

[fsn31101-bib-0003] Chen, L.‐S. , Ma, Y. , Chen, L.‐J. , Zhao, C.‐H. , Maubois, J.‐L. , Jiang, T.‐M. , … He, S.‐H. (2010). Antioxidant activity of two yeasts and their attenuation effect on 4‐nitroquinoline 1‐oxide induced in vitro lipid peroxidation. International Journal of Food Science & Technology, 45(3), 555–561.

[fsn31101-bib-0004] Cho, Y.‐J. , Kim, D.‐H. , Jeong, D. , Seo, K.‐H. , Jeong, H. S. , Lee, H. G. , & Kim, H. (2018). Characterization of yeasts isolated from kefir as a probiotic and its synergic interaction with the wine byproduct grape seed flour/ extract. LWT‐ Food Science and Technology, 90, 535–539. 10.1016/j.lwt.2018.01.010

[fsn31101-bib-0005] Datta, S. , Annapore, U. S. , & Timson, D. J. (2017). Different specificities of two aldehyde dehydrogenases from *saccharomyces cerevisiae* var boulardii. Bioscience Reports, 37(2), BSR20160529. 28126723 10.1042/BSR20160529 PMC5483954

[fsn31101-bib-0006] Devlieghere, F. , Vermeiren, L. , & Debevere, J. (2004). New preservation technologies: Possibilities and limitations. International Dairy Journal, 14, 273–285. 10.1016/j.idairyj.2003.07.002

[fsn31101-bib-0007] Farvin, K. H. S. , Grejsen, H. D. , & Jacobsen, C. (2012). Potato peel extract as a natural antioxidant in chilled storage of minced horse mackerel (*Trachurus trachurus*): Effect on lipid and protein oxidation. Food Chemistry, 131, 843–851. 10.1016/j.foodchem.2011.09.056

[fsn31101-bib-0008] Fauconnier, M.‐L. , Mpambara, A. , Delcarte, J. , Jacques, P. , Thonart, P. , … Marlier, M. (1999). Conversion of green note aldehydes into alcohols by yeast alcohol dehydrogenase. Biotechnology Letters, 21(7), 629–633.

[fsn31101-bib-0009] Foo, J. L. , Susanto, A. V. , Keasling, J. D. , Leong, S. S. J. , & Chang, M. W. (2017). Whole‐cell biocatalytic and de novo production of alkanes from free fatty acids in *Saccharomyces cerevisiae* . Biotechnology and Bioengineering, 114(1), 232–237. 26717118 10.1002/bit.25920 PMC5132040

[fsn31101-bib-0010] Fu, X. , Hayat, K. , Li, Z. , Lin, Q. , Xu, S. , & Wang, S. (2012). Effect of microwave heating on the low‐salt gel from silver carp (*Hypophthalmichthys molitrix*) surimi. Food Hydrocolloids, 27(2), 301–308. 10.1016/j.foodhyd.2011.09.009

[fsn31101-bib-0011] Fu, X. , Lin, Q. , Xu, S. , & Wang, Z. (2015). Effect of drying methods and antioxidants on the flavor and lipid oxidation of silver carp slices. LWT ‐ Food Science and Technology, 61, 251–257. 10.1016/j.lwt.2014.10.035

[fsn31101-bib-0012] Fu, X. , Xu, S. , & Wang, Z. (2009). Kinetics of lipid oxidation and off‐odor formation in silver carp mince: The effect of lipoxygenase and hemoglobin. Food Research International, 42, 85–90. 10.1016/j.foodres.2008.09.004

[fsn31101-bib-0013] Geng, S. R. , Xia, H. Z. , Zu, X. Y. , Chen, Y. X. , Ye, L. X. , & Xiong, G. Q. (2014). Analysis of volatile compounds from irradiated yeast extract by headspace solid phase micro‐extraction coupled with gas chromatography‐mass spectrometry. Food Science, 35(6), 55–59.

[fsn31101-bib-0014] Kakuta, T. , Hoshikuma, A. , Kanauchi, M. , Shindoh, H. , Yoshizawa, K. , & Koizumi, T. (1999). Studies on antioxidant produced by the yeast. Part I. Identification and some properties of antioxidant produced by the yeast. Food Preservation Science, 25, 215–220. 10.5891/jafps.25.215

[fsn31101-bib-0015] Lehto, S. , Laakso, S. , & Lehtinen, P. (2003). Enzymatic oxidation of hexanal by oat. Journal of Cereal Science, 38, 199–203. 10.1016/S0733-5210(03)00028-6

[fsn31101-bib-0016] Li, G. , Sinclair, A. J. , & Li, D. (2011). Comparison of lipid content and fatty acid composition in the edible meat of wild and cultured freshwater and marine fish and shrimps from China. Journal of Agricultural and Food Chemistry, 59, 1871–1881. 10.1021/jf104154q 21291233

[fsn31101-bib-0017] Lynch, M. P. , & Faustman, C. (2000). Effect of aldehyde lipid oxidation products on myoglobin. Journal of Agricultural and Food Chemistry, 48, 600–604. 10.1021/jf990732e 10725121

[fsn31101-bib-0018] Munasinghe, D. M. , Ichimaru, K.‐I. , Ryuno, M. , Ueki, N. , Matsui, T. , Sugamoto, K. , … Sakai, T. (2003). Lipid peroxidation‐derived hepatotoxic aldehydes, 4‐hydroxy‐2E‐hexenal in smoked fish meat products. Fisheries Science, 69(1), 189–194. 10.1046/j.1444-2906.2003.00605.x

[fsn31101-bib-0019] Papastergiadis, A. , Fatouh, A. , Jacxsens, L. , Lachat, C. , Shrestha, K. , Daelman, J. , … DeMeulenaer, B. (2014). Exposure assessment of malondialdehyde, 4‐Hydroxy‐2‐(E)‐Nonenal and 4‐Hydroxy‐2‐(E)‐ Hexenal through specific foods available in Belgium. Food and Chemical Toxicology, 73, 51–58. 25035169 10.1016/j.fct.2014.06.030

[fsn31101-bib-0020] Qin, N. , Li, D. , Hong, H. , Zhang, Y. , Zhu, B. , & Luo, Y. (2016). Effects of different stunning methods on the flesh quality of grass carp (*Ctenopharyngodon idellus* ) fillets stored at 4°C. Food Chemistry, 201, 131–138. 26868557 10.1016/j.foodchem.2016.01.071

[fsn31101-bib-0021] Richards, M. P. , & Hultin, H. O. (2000). Effect of pH on lipid oxidation using trout hemolysate as a catalyst: A possible role for deoxyhemoglobins. Journal of Agricultural & Food Chemistry, 48, 3141–3147. 10956082 10.1021/jf991059w

[fsn31101-bib-0022] Sakai, T. , Matsushita, Y. , Sugamoto, K. , & Uchida, K. (1997). Lipid peroxidation‐derived hepatotoxic aldehyde, 4‐Hydroxy‐2‐hexenal, in fish. Bioscience Biotechnology and Biochemistry, 61(8), 1399–1400.

[fsn31101-bib-0023] Sawada, H. , Hara, A. , Nakayama, T. , & Hayashibara, M. (1982). Kinetic mechanisms in the reduction of aldehydes and ketones catalyzed by rabbit liver aldehyde reductases and hydroxysteroid dehydrogenases. Journal of Biochemistry, 92(1), 185–191. 6749832 10.1093/oxfordjournals.jbchem.a133915

[fsn31101-bib-0024] Shi, C. E. , Cui, J. , Yin, X. , Luo, Y. , & Zhou, Z. (2014). Grape seed and clove bud extracts as natural antioxidants in silver carp (*Hypophthalmichthys molitrix*) fillets during chilled storage: Effect on lipid and protein oxidation. Food Control, 40, 134–139. 10.1016/j.foodcont.2013.12.001

[fsn31101-bib-0025] Song, Y. , Liu, L. , Shen, H. , You, J. , & Luo, Y. (2011). Effect of sodium alginate‐based edible coating containing different anti‐oxidants on quality and shelf life of refrigerated bream (*Megalobrama amblycephala*). Food Control, 22, 608–615. 10.1016/j.foodcont.2010.10.012

[fsn31101-bib-0026] Steppeler, C. , Haugen, J. E. , Rødbotten, R. , & Kirkhus, B. (2016). Formation of malondialdehyde, 4‐Hydroxynonenal, and 4‐Hydroxyhexenal during in vitro digestion of cooked beef, pork, chicken and salmon. Journal of Agricultural & Food Chemistry, 64(2), 487–496. 26654171 10.1021/acs.jafc.5b04201

[fsn31101-bib-0027] Surh, J. , & Kwon, H. (2005). Estimation of daily exposure to 4‐hydroxy‐2‐alkenals in Korean foods containing n‐3 and n‐6 polyunsaturated fatty acids. Food Additives & Contaminants, 22(8), 701–708. 16147425 10.1080/02652030500164359

[fsn31101-bib-0028] Svitel, J. , & Sturdik, E. (1995). n‐Propanol conversion to propionic acid by gluconobacter oxydans. Enyzme and Microbial Technology, 17, 546–550. 10.1016/0141-0229(94)00088-9

[fsn31101-bib-0029] Tanaka, R. , Naiki, K. , Tsuji, K. , Nomata, H. , Sugiura, Y. , Matsushita, T. , & Kimura, I. (2013). Effect of antioxidative treatments on lipid oxidation in skinless fillet of pacific saury *Cololabis saira* in frozen storage. Journal of Food Processing and Preservation, 37, 325–334.

[fsn31101-bib-0030] Taskaya, L. , Chen, Y. , & Jaczynski, J. (2010). Color improvement by titanium dioxide and its effect on gelation and texture of proteins recovered from whole fish using isoelectric solubilization/precipitation. LWT‐Food Science and Technology, 43(3), 401–408. 10.1016/j.lwt.2009.08.021

[fsn31101-bib-0031] van Iersel, M. , Brouwer–Post, E. , Rombouts, F. M. , & Abee, T. (2000). Influence of yeast immobilization on fermentation and aldehyde reduction during the production of alcohol‐free beer. Enzyme and Microbial Technology, 26(8), 602–607. 10.1016/S0141-0229(00)00140-X 10793207

[fsn31101-bib-0032] Vieira, E. F. , Melo, A. , & Ferreira, I. M. P. L. V. O. (2017). Autolysis of intracellular content of Brewer’s spent yeast to maximize ACE‐inhibitory and antioxidant activities. LWT ‐ Food Science and Technology, 82, 255–259. 10.1016/j.lwt.2017.04.046

[fsn31101-bib-0033] Wang, H.‐Y. , Xiao, D.‐F. , Zhou, C. , Wang, L.‐L. , Wu, L. , Lu, Y.‐T. , … Ma, M.‐G. (2017). YLL056C from *Saccharomyces cerevisiae* encodes a novel protein with aldehyde reductase activity. Applied Microbiology and Biotechnology, 101(11), 4507–4520. 10.1007/s00253-017-8209-5 28265724

[fsn31101-bib-0034] Wang, Y. , Wu, Y. , Li, L. , Yang, X. , Wang, X. Cai, Q. , … Wei, Y. (2017). Effect of antioxidant lactic acid Bacteria on lipid hydrolysis and oxidation of dry‐cured hairtail: A study using principal components analysis. Food Science, 38(8), 231–238.

[fsn31101-bib-0035] Yao, S. , Ye, Y. , Cai, C. , Zhang, B. , Xiao, P. , Chen, K. , & Peng, K. (2014). Protective effect of water soluble material of yeast culture on malondialdehyde damaged intestinal mucosal cells in vitro of grass carp (*Ctenopharyngodon idella*). Chinese Journal of Animal Nutrition, 26(9), 2652–2663.

[fsn31101-bib-0036] Zeng, X. , Xia, W. , Jiang, Q. , & Yang, F. (2013). Effect of autochthonous starter cultures on microbiological and physico‐chemical characteristics of Suan Yu, a traditional chinese low salt fermented fish. Food Control, 33(2), 344–351. 10.1016/j.foodcont.2013.03.001

[fsn31101-bib-0037] Zhou, X. , Chong, Y. , Ding, Y. , Gu, S. , & Liu, L. (2016). Determination of the effects of different washing processes on aroma characteristics in silver carp mince by MMSE–GC–MS, e‐nose and sensory evaluation. Food Chemistry, 207, 205–213. 10.1016/j.foodchem.2016.03.026 27080898

[fsn31101-bib-0038] Zhu, J. C. , Wang, L. Y. , Xiao, Z. B. , & Niu, Y. W. (2018). Characterization of the key aroma compounds in mulberry fruits by application of gas chromatography–olfactometry (GC‐O), odor activity value (OAV), gas chromatography‐mass spectrometry (GC–MS) and flame photometric detection (FPD). Food Chemistry, 245, 775–785. 10.1016/j.foodchem.2017.11.112 29287440

